# A Possible Role for HSV-1-Specific Humoral Response and *PILRA* rs1859788 Polymorphism in the Pathogenesis of Parkinson’s Disease

**DOI:** 10.3390/vaccines9070686

**Published:** 2021-06-22

**Authors:** Simone Agostini, Roberta Mancuso, Andrea S. Costa, Lorenzo A. Citterio, Franca R. Guerini, Mario Meloni, Jorge Navarro, Mario Clerici

**Affiliations:** 1 IRCCS Fondazione Don Carlo Gnocchi ONLUS, 20148 Milan, Italy; rmancuso@dongnocchi.it (R.M.); acosta@dongnocchi.it (A.S.C.); lcitterio@dongnocchi.it (L.A.C.); fguerini@dongnocchi.it (F.R.G.); mmeloni@dongnocchi.it (M.M.); jnavarro@dongnocchi.it (J.N.); mario.clerici@unimi.it (M.C.); 2 Department of Pathophysiology and Transplantation, University of Milan, 20122 Milan, Italy

**Keywords:** Parkinson’s disease, HSV-1, paired immunoglobulin-like type 2 receptor alpha, antibody titers, rehabilitation

## Abstract

The etiology of Parkinson’s disease (PD), a progressive nervous system disorder that affects movement, is still unknown; both genetic and environmental factor are believed to be involved in onset of the disease and its development. Herpes simplex virus type 1 (HSV-1), in particular, is suspected to have a role in PD. Paired Immunoglobulin-like type 2 receptor alpha (PILRA) is an inhibitory receptor that down-regulates inflammation and is expressed on innate immune cells. The *PILRA* rs1859788 polymorphism is protective against Alzheimer’s disease, even in relation with HSV-1 antibody titers, but no data are available in PD. We analyzed HSV-1 antibody titers and *PILRA* rs1859788 in PD (*n =* 51) and age-and sex-matched healthy controls (HC; *n =* 73). Results showed that HSV-1, but not cytomegalovirus (CMV) or human herpes virus type 6 (HHV-6) antibody titers were significantly higher in PD compared to HC (*p* = 0.045). The rs1859788 polymorphism was not differentially distributed between PD and HC, but the minor allele A was more frequently carried by PD (68%) compared to HC (50%) (*p* = 0.06). Notably, the rs1859788 minor allele A was statically more frequent in male PD (65%) compared to male HC (37%) (*p* = 0.036). Finally, no relation was found between HSV-1 antibody titers and *PILRA* genotype. Results herein suggest an involvement of HSV-1 in PD and indicate a possible interaction between *PILRA* gene polymorphisms and this neuropathology.

## 1. Introduction

Parkinson’s disease (PD), one of the most common neurodegenerative conditions, affects about 2% of older individuals and about 6 million individuals worldwide, and is a progressive nervous system disorder that impairs movement [1]. The most common PD symptoms are resting tremors, unstable posture, bradykinesia, rigidity, and non-motor symptoms, such as dysphagia [2]. PD is characterized by the loss of neurons in the substantia nigra, possibly resulting from the accumulation of misfolded and aggregated forms of α-synuclein protein within Lewy bodies [3,4]. α-synuclein is a 140 aa protein located at presynaptic terminals. Its exact role is not completely clear, but it seems that can regulate the amount of SNARE complex, which regulates the neurotransmitters release [5].

Microglia-driven chronic neuroinflammation, oxidative stress, autophagy disruption, and mitochondrial dysfunction are typical features of the pathology [1,6,7].

Currently there is no cure for the disease, but dopaminergic therapies as levodopa and dopamine agonists are commonly used for the control of motor symptoms.

The etiology of PD is still unknown, but epidemiological studies suggest a strong association between genetics and environmental factors in the onset and development of the disease [8]. Thus, several viruses have been associated with the disease: influenza A, measles, hepatitis C virus, and herpesviruses [9,10,11].

A possible role for viral infections in the development of PD was initially proposed by Von Economo [12]. This researcher observed that the lethargic encephalitis, appearing as a complication of the Spanish Flu pandemic of 1918, was associated with brain inflammatory lesions in the midbrain tegmentum and the substantia nigra. Although the mechanistic link between influenza and PD remains enigmatic and controversial, mouse model showed that highly pathogenic strain H5N1 influenza can reach the brain and initiate central nervous system (CNS) disorders of protein aggregation including Parkinson’s and Alzheimer’s diseases [13].

Among herpesviruses, in particular, human herpes simplex virus type 1 (HSV-1) is strongly suspected to have a role in the disease. Thus, high HSV-1-specific antibody titers and a more frequent incidence of HSV-1 infections were observed in patients with idiopathic PD [14,15,16,17,18], although these findings were not always confirmed [19].

HSV-1 is a double-stranded DNA virus that belongs to the *Alphaherpesvirinae* subfamily and commonly infects humans [20]. After the primary infection, usually occurring before adulthood, HSV-1 can establish latency in sensory ganglia; over time viral reactivations are observed, but they are controlled by the host immune response [21]. HSV-1 is a highly prevalent infection worldwide, as about the 67% of the population under the age of 50 was shown to be infected [22]. Some results suggest that if the equilibrium between viral reactivation and host immune response is lost, excessive HSV-1 replication, together with neuroinflammation become key factors in the pathogenesis of PD [23,24,25]. This hypothesis fits within the dual-hit theory for PD, which postulates that the initial event of the disease is a pathogenic access of viruses into the brain through the stomach and the nose [26]: the observation that HSV-1 can establish life-long persistence in the olfactory bulb reinforces a possible pathogenic role for this virus in PD [27,28].

HSV-1 in not suggested to only be involved in PD, but is also suspected to participate to the pathogenesis of Alzheimer’s disease (AD). Thus: (1) HSV-1 specific IgG titers and avidity are higher in AD patients compared to healthy controls [29,30]; (2) HSV-1 antibody avidity associates with AD conversion in individuals with mild cognitive impairment (MCI) [31]; (3) HSV-1 specific IgG titers positively correlate with brain grey matter volumes and cortical thinning in AD patients [30,32]; (4) in vitro and animal studies showed that HSV-1 can cause accumulation of amyloid-beta and hyperphosphorylated tau, key factors of the disease [33,34,35]; (5) an in vitro study on human neural cells showed that HSV-1 directly impairs autophagy, reducing amyloid-beta degradation [36].

Among herpesviruses, even cytomegalovirus (CMV) was suspected to have a role in PD [37], at least in association with other viral or bacterial infection [38], whereas for the neurotropic human herpes virus 6 (HHV-6), to our knowledge, in scientific literature, just a case report presented a possible relation with Parkinsonism symptoms [39].

As is the case for PD, the pathogenesis of AD includes the presence of barely identified genetic factors as well; one of these factors is the polymorphisms of paired Immunoglobulin-like type 2 receptor alpha (PILRA), an inhibitory receptor expressed on innate immune cells that down-regulates inflammation [40,41]. The human *PILRA* gene, with a length of 32,567 nucleotides, is located on chromosome 7 (7q22.1), and coded for a 303 amino acids (aa) protein, characterized by 3 different domains: an extracellular domain (178 aa), a transmembrane domain (21 aa), and a cytoplasmatic domain (85 aa). The gene includes a number of single nucleotide polymorphisms (SNP), amongst them the rs1859788 causes an A-to-G transition, and a consequent Arginine (R)-to-Glycin (G) substitution at 78 amino acid (aa).

This SNP was shown to be an AD risk locus [42], and recently it was demonstrated that the presence of Arginine at 78 aa (R78) is protective against AD development [43]. PILRA plays a key role in HSV-1 infection, as the virus binds this protein to infect cells [44]. Notably, the PILRA R78 SNP reduces viral infectivity; as a consequence, R78/R78 people are less susceptible to HSV-1 infection and, in case of infection, HSV-1 recurrence and reactivation are greatly reduced [43]. These results were further confirmed by recent data indicating the presence of a correlation between the *PILRA* rs1859788 polymorphism and HSV-1 specific IgG titers in AD [45].

Starting from this background, we verified these parameters in PD patients in the attempt to shed light on the pathogenesis of this disease.

## 2. Materials and Methods

### 2.1. Patients and Controls

A total of 124 individuals were included in the study: 51 (15 male and 36 female) patients with a diagnosis of Parkinson’s disease (PD) and 73 (32 male and 41 female) age-and sex-matched healthy controls (HC). All subjects were recruited by the IRCCS Santa Maria Nascente, Don Gnocchi Foundation, Milan, Italy and were enrolled in rehabilitation programs. Patients were diagnosed as being affected by PD by clinical evaluation, according to the Movement Disorder Society (MDS) Clinical Diagnostic Criteria for PD [46]. Disease severity, based on the Modified Hoehn and Yahr (H&Y) stage and MDS-Unified Parkinson’s Disease Rating Scale-III (MDS-UPDRS-III) scores [47], as well as dopaminergic and non-dopaminergic antiparkinsonian therapy were collected for each patient. Levodopa equivalent daily dose (LEDD) was calculated for each patient [48]. The study conformed the ethical principles of the Declaration of Helsinki; all subjects gave informed and written consent according to a protocol approved by the local ethics committee of the Don Carlo Gnocchi Foundation (#06_21/06/2018). Whole blood and serum samples were collected from all the enrolled subjects.

### 2.2. ELISA

#### 2.2.1. α-Synuclein Measurement

Soluble α-synuclein was measured in plasma of all the enrolled subjects by using a commercial enzyme-linked immunosorbent assay (ELISA), according to the manufacturer’s instructions (IBL International, Hamburg, German). Briefly, 100 μL of plasma samples diluted (1:2) with sample diluent were transferred into the pre-coated microwells and the plates were incubated overnight at 4 °C. After washing steps with washing buffer, 100 μL of labeled antibody were added to each well and incubated for 60 min at 4 °C. After re-washing step, 100 μL of chromogen solution were added to each well and incubated at room temperature for 30 min. Finally, 100 μL of stop solution were added to each well and the reaction stopped. The wells were read on a plate reader (Sunrise, Tecan, Mannedorf, Switzerland) and optical densities (OD) of wells were determined at 450 nm. α-synuclein concentration was expressed as ng/mL (sensitivity: 0.03 ng/mL).

#### 2.2.2. Anti-Herpetic IgG Antibody Measurements

HSV-1, CMV, and HHV-6 total IgG titers were measured in serum of all the enrolled subjects. The viral IgG titers were used by ELISA (HSV-1 IgG, IBL International, Hamburg, Germany, CMV IgG, Abcam, Cambridge, MA. US, and HHV-6 IgG, Abnova, Taipei, Taiwan) according to standard protocol. Briefly, 100 μL of serum samples diluted (1:100 for HSV-1 and CMV, 1:10 for HHV-6) with appropriate sample diluent were transferred into the appropriate viral antigens coated polystyrene microwells and the plates were incubated for 60 min (at room temperature for HHV-6, at 37 °C for CMV and HSV-1). After washing steps with washing buffer to remove the unbound proteins, 100 μL of appropriate peroxidase conjugate was added to each well and incubated for 60 min for HHV-6, for 30 min for CMV and HSV-1 (at room temperature for HHV-6 and HSV-1, at 37 °C for CMV). After re-washing step, 100 μL of appropriate chromogen/substrate solution were added to each well and incubated at room temperature for 15 min for HSV-1 and CMV, 20 min for HHV-6. Finally, 100 μL of stop solution were added to each well and the reaction stopped. The wells were read on a plate reader (Sunrise, Tecan, Mannedorf, Switzerland) and optical densities (OD) of wells were determined at 450/620 nm. HSV-1 Ab titers are expressed as units (U), HHV-6 Ab titers were expressed as positivity index (PI), and CMV Ab titers were expressed as arbitrary units/mL (AU/mL), as indicated by the ELISA kits. For HSV-1, subjects with U ≥ 5 were considered seropositive; for CMV, subjects with U/mL ≥10 were considered seropositive; for HHV-6, subjects with PI ≥ 1.11 were considered seropositive.

HSV-1 IgG avidity was measured using a protein-denaturing agent, as previously described [31]. Briefly, the protocol used is the protocol for ELISA analysis, with the addition of 6 M urea to the washing solution at the washing step after serum reaction. The avidity index (indicated as %) was calculated as follows: anti-HSV-1 Ab titer measured with washing including urea/anti-HSV-1 Ab titer measured with washing without urea.

### 2.3. PILRA rs1859788 Genotyping

Genomic DNA was isolated from whole blood by phenol-chloroform extraction for all the enrolled population. Custom-design TaqMan probes were used for the *PILRA* rs1859788 genotyping, as reported in our previous study [45], using Bio-Rad CFX96 Touch^TM^ Real-Time PCR Detection System (Bio-Rad. Hercules, CA, USA). Primers and probes are: Forward primer: 5′-GCT CCC GAC GTG AGA ATA TCC-3′; Reverse primer: 5′-GCG GCC TTG TGT AGA A-3′; Reporter 1 sequence: HEX 5′-ACT TCC ACG GGC AGT C-3′-MGBEQ; Reporter 2 sequence: FAM 5′-ACT TCC ACA GGC AGT C-3′-MGBEQ. The PCR amplification was performed as follows: 2 min at 50 °C, 10 min at 95 °C, 40 cycles consisting of 15 s denaturation at 95 °C and a 60 s annealing and extension at 60 °C, followed by a hold at 4 °C.

### 2.4. Statistical Analysis

Chi-square goodness of fit test was used to verify that the rs1859788 polymorphism was in Hardy-Weinberg (HW) equilibrium and to evaluate case-control differences of SNP distribution. Normally distributed data were summarized as mean ± standard deviation, and comparison among groups were analyzed by Student t test. Not-normally distributed data were summarized as median and interquartile range (IQR: 25th and 75th percentile), and comparisons were analyzed by Mann-Whitney U test, as appropriate. Qualitative data were compared using Fisher’s exact test and chi-squared test and *p*-value was considered significant when ≤0.05 after Bonferroni correction for 2 degrees of freedom (p_c_) in 2 × 3 and 2 × 2 contingency tables. The statistical analyses were accomplished using commercial software (MedCalc Statistical Software version 14.10.2., Ostend, Belgium, and IBM SPSS Statistics 26.0, IBM Inc. Chicago, IL, USA). *p*-values ≤ 0.05 were considered statistically significant.

## 3. Results

### 3.1. Clinical Characterization of the Study Population

Demographic and clinical characteristic of the individuals enrolled in the study are summarized in Table 1. Briefly, gender and age were comparable between PD and HC; the disease duration of PD patients was 7.4 ± 5.1 years and total LEDD was 512.8 ± 280.8 mg/die. The majority of patients reported anosmia or constipation in the initial stages of the disease.

### 3.2. Plasmatic α-Synuclein Concentration

The α-synuclein was detected in plasma of all patients and controls. It was, as expected, significantly higher in PD (19.43 ng/mL; 15.22–23.12 ng/mL) compared to HC (12.25 ng/mL; 8.00–20.20 ng/mL; *p* = 0.0001). No correlation was found between α-synuclein concentration and demographic (age and gender) and clinical data (disease duration, MDP-UPDRS III, modified H&Y, LEDD).

### 3.3. Virological Results

The overall rate of seropositivity was 94% for HSV-1, 93% for CMV, and 62% for HHV-6 (both CMV and HHV-6 were used as controls for HSV-1 seroprevalence). HSV-1 seropositivity was higher in PD (98%) than HC (92%), whereas for the other two herpesviruses an opposite situation was observed as antibodies for both CMV (PD: 88%; HC: 98%) and HHV-6 (PD: 56%; HC: 67%) were more frequently observed in HC than in PD patients.

**Figure 1:**
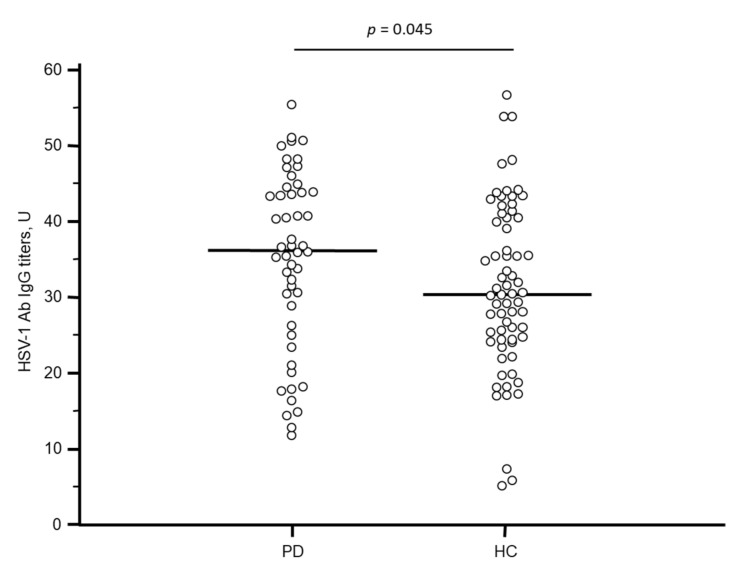
Distribution of serum HSV-1-specific IgG titers in Parkinson’s Disease patients and Healthy Controls. Horizontal lines represent the median. PD: Parkinson’s Disease; HC: Healthy Controls; U: units.

HSV-1 antibody titers levels were significantly higher in PD compared to HC (*p* = 0.045) (Figure 1); the HSV-1 avidity index was increased as well, although not significantly, in PD compared to HC. In contrast with these results, no significant difference was observed regarding the antibody titers of CMV and HHV-6 between the two groups. All the virological data are schematized in Table 2. Finally, no correlations were observed among HSV-1, CMV, HHV-6 antibody titers, HSV-1 avidity index, plasmatic α-synuclein concentration, and demographic (age and gender) and clinical data (disease duration, MDP-UPDRS III, modified H&Y, LEDD) (data not shown).

### 3.4. PILRA rs1859788 Genotyping

The genotype distribution of *PILRA* (A/G) rs1859788 polymorphism was in Hardy-Weinberg (HW) equilibrium both in PD and HC groups. Although *PILRA* rs1859788 was not differentially distributed between PD and HC, a trend of significance (*p* = 0.06) was found regarding the minor allele A (AA + AG), that was more frequently carried by PD (67.3%) compared to HC (50%) (Table 3).

**Figure 2:**
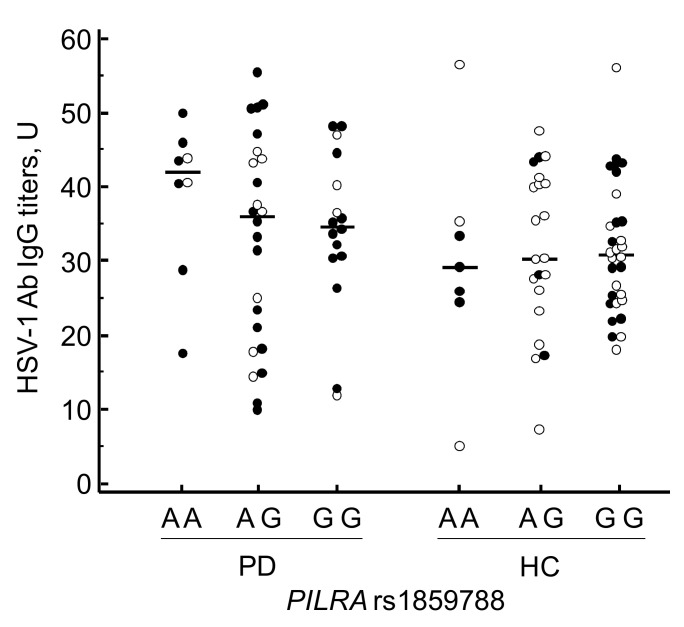
Distribution of serum HSV-1-specific IgG titers in Parkinson’s Disease patients and Healthy Controls according to *PILRA* rs2859788 genotypes and gender. Black dots: male; white dots: female. Horizontal lines represent the median. PD: Parkinson’s Disease; HC: Healthy Controls; U: units.

Moreover, when the study population was split in relation with gender, the rs1859788 genotypes distribution was statistically different in male PD compared to male HC (*p*_c_ = 0.05; d.f.: 2; χ^2^ = 5.99), with, again, the minor allele A (AA + AG) being statically more frequent in male PD (64.7%) compared to male HC (37.1%) (*p*_c_ = 0.036; OR: 3.056; 95% CI: 1.07–9.09) (Table 3). No relations were observed among *PILRA* genotype and HSV-1 (Figure 2), CMV and HHV-6 titers, HSV-1 avidity index and plasmatic α-synuclein concentration (data not shown).

## 4. Discussion

The pathogenesis of PD is only barely understood. Among several factors—host immune system, host genetics, environmental factors—pathogens are suspected to have a possible role in the disease. Among pathogens, HSV-1, in particular is strongly suspected to be associated with the disease.

Here we show that higher HSV-1-specific antibody titers are seen in PD patients compared to HC, and that this is specific for HSV-1, as the antibody titers against other neurotropic viruses, including, CMV and HHV-6, another neurotropic herpesvirus, were similar in PD and HC. How HSV-1 may contribute to the disease is not understood. One hypothesis is that HSV-1 could alter the host innate immunity, and, in particular, could impair the INF-β-mediated immune response [49]. Although an altered immune response has not been associated with the initial events that lead to PD, an alteration in the production of pro-inflammatory cytokines was repeatedly shown to facilitate disease progression through the involvement of microglia and astrocytes.

Parkinson’s disease was recently hypothesized to be an autoimmune disease, or at least a disease with features typical of autoimmunity [50,51]. This idea stems from the observation that the accumulation of misfolded and aggregated forms of α-synuclein can activate the adaptive immune response [52,53].

HSV-1 could also have a role in triggering autoimmunity in PD, as regions of molecular mimicry that could result in the stimulation of an immunologic cross-reactivity between HSV-1 and human α-synuclein do exists [23]. In this regard it is interesting to underline that both α-synuclein_100–114_ and HSV-1 UL4222-36 peptides can stimulate in vitro cell-mediated immune responses by peripheral blood mononuclear cells (PBMCs) of PD patients [24].

In our study population, high concentration of plasmatic α-synuclein was found in PD patients, confirming previous results [54,55,56]. We did not observe a relation between α-synuclein concentration and HSV-1-specific antibody in our patients, possibly because of the limited dimension of the study population, other studies in ampler cohorts are needed to verify if a direct association exists between these two parameters.

PD patients were also characterized by having a higher frequency of the *PILRA* rs1859788 minor allele A (AA + AG), this effect was even more evident when data were analyzed using sex as a variable. The presence of A allele causes the Glycin-to-Arginin substitution at 78 aa (G78R) of PILRA protein. If on the one hand this substitution seems to decrease HSV-1 infectivity [43], as the virus usually binds PILRA to enter cells [44], on the other hand the *PILRA* G/A substitution was suggested to interfere with the ability of PILRA to reduce microglial activation via PILRB/DA12 signaling [40,41,43,57]. PILRA regulates not only microglia and monocytes or macrophages [34], but also other immune cells, as neutrophils [40], it is thus tempting to speculate that an increased presence of PILRA G78R variant in PD patients could explain the increase inflammation status of these patients, and, in particular, the autoimmunity features that characterize the disease. Shedding light on the possible interactions between the humoral immune response against HSV-1 and other common pathogens and the genetics in PD could help understanding the possible impact of infections on disease onset and progression.

## 5. Conclusions

In conclusion, although preliminary and needing confirmation in bigger cohorts of patients, results herein could support a possible involvement of HSV-1 in Parkinson’s disease, and may suggest that polymorphisms of the *PILRA* gene—a gene involved in HSV-1 infection—play a role in the neuroinflammation that accompanies this disease. The nature of HSV-1-specific immune responses will also need to be investigated in more depth in PD patients. In particular, the characterization of IgG subclasses and of the neutralizing activity of HSV-1-specific antibodies will need to be analyzed in PD to better understand the presence of possible interactions between HSV-1 infection and the disease.

## Figures and Tables

**Table 1:** Demographic and clinical characteristics of the individuals enrolled in the study.

Demographic and Clinical Characteristics	PD Patients	Healthy Controls
*n.*	51	73
Gender (M:F)	15:36	32:41
Age, years (mean ± SD)	69.5 ± 8.5	69.5 ± 8.0
Disease duration (mean ± SD)	7.4 ± 5.1	---
MDS-UPDRS III (mean ± SD)	27.6 ± 13.0	---
Modified H&Y (median; IQR)	2.0; 1.5–3.0	---
LEDD, mg/die (mean ± SD)	512.8 ± 280.8	---
α-synuclein, ng/mL (median, IQR)	19.43; 15.22–23.12 ^1^	12.25; 8.00–20.20

^1^ PD vs. HC: *p* = 0.0001. PD: Parkinson’s disease; M: male; F: female; n.: absolute number; SD: standard deviation; IQR: Interquartile Range; MDS-UPDRS III: Movement Disorders Society Unified Parkinson’s Disease Rating Scale; Modified H&Y: Modified Hoehn and Yahr scale; LEDD: levodopa equivalent daily dose.

**Table 2:** Virological characteristics of the individuals enrolled in the study.

Virological Characteristics	PD Patients	Healthy Controls
HSV-1 seropositivity, %	98	92
HSV-1 IgG, U (median; IQR)	36.2; 32.8–40.6 ^1^	30.3; 27.7–34.7
HSV-1 avidity index, % (median; IQR)	92.1; 83.4–97.5	88.6; 83.8–91.8
CMV seropositivity, %	88	98
CMV IgG, AU/mL (median; IQR)	161.2; 141.7–196.9	193.5; 159.0–221.2
HHV-6 seropositivity, %	56	67
HHV-6, P.I. (median; IQR)	5.4; 1.7–2.5	2.6; 2.0–3.3

^1^ PD vs. HC: *p* = 0.045. PD: Parkinson’s disease; IQR: Interquartile Range; U: units; AU/mL: arbitrary unit/mL; P.I.: positivity index; HSV-1: herpes simplex virus type 1; CMV: cytomegalovirus; HHV-6: human herpesvirus type 6; IgG: immunoglobulin G.

**Table 3:** Genotype and allelic distribution of *PILRA* rs1859788 polymorphism in the individuals enrolled in the study.

Groups	*PILRA* rs1859788			*PILRA* rs1859788		*PILRA* rs1859788	
Genotype/Allele	A G	A G	G G	A (A + AG)	G G	A	G
PD, %	16.3	51.0	32.7	67.3	32.7	41.8	58.2
HC, %	12.5	37.5	50.0	50.0	50.0	31.3	68.7
**Men**							
PD, % ^1^	17.7	47.0	35.3	64.7 ^2^	35.3	41.2	58.8
HC, %	18.5	18.5	63.0	37.1	62.9	27.8	72.2
**Women**							
PD, %	13.3	60.0	26.7	73.3	26.7	43.3	56.7
HC, %	8.1	51.4	40.5	59.5	40.5	33.8	66.2

^1^ PD vs. HC: p_c_ = 0.05; d.f.: 2; Chi^2^ = 5.99. ^2^ PD vs. HC: p_c_ = 0.0036; OR: 3.056; 95% CI = 1.07–9.09. PD: Parkinson’s disease; HC: Healthy Controls; PILRA: paired immunoglobulin-like type 2 receptor alpha; d.f.: degrees of freedom; OR: odd ratio; CI: confidence interval.

## Data Availability

The data presented in this study are available on request from the corresponding author.
